# BETA: A Large Benchmark Database Toward SSVEP-BCI Application

**DOI:** 10.3389/fnins.2020.00627

**Published:** 2020-06-23

**Authors:** Bingchuan Liu, Xiaoshan Huang, Yijun Wang, Xiaogang Chen, Xiaorong Gao

**Affiliations:** ^1^Department of Biomedical Engineering, Tsinghua University, Beijing, China; ^2^State Key Laboratory on Integrated Optoelectronics, Institute of Semiconductors, Chinese Academy of Sciences, Beijing, China; ^3^Institute of Biomedical Engineering, Chinese Academy of Medical Sciences and Peking Union Medical College, Tianjin, China

**Keywords:** brain-computer interface (BCI), steady-state visual evoked potential (SSVEP), electroencephalogram (EEG), public database, frequency recognition, classification algorithms, signal-to-noise ratio (SNR)

## Abstract

The brain-computer interface (BCI) provides an alternative means to communicate and it has sparked growing interest in the past two decades. Specifically, for Steady-State Visual Evoked Potential (SSVEP) based BCI, marked improvement has been made in the frequency recognition method and data sharing. However, the number of pubic databases is still limited in this field. Therefore, we present a **BE**nchmark database **T**owards BCI **A**pplication (BETA) in the study. The BETA database is composed of 64-channel Electroencephalogram (EEG) data of 70 subjects performing a 40-target cued-spelling task. The design and the acquisition of the BETA are in pursuit of meeting the demand from real-world applications and it can be used as a test-bed for these scenarios. We validate the database by a series of analyses and conduct the classification analysis of eleven frequency recognition methods on BETA. We recommend using the metric of wide-band signal-to-noise ratio (SNR) and BCI quotient to characterize the SSVEP at the single-trial and population levels, respectively. The BETA database can be downloaded from the following link http://bci.med.tsinghua.edu.cn/download.html.

## 1. Introduction

The brain-computer interface (BCI) provides a new way for brain interaction with the outside world, and it is based on measuring and converting brain signals to the external commands without involving the peripheral nervous system (Wolpaw et al., 2002). The BCI technology has considerable scientific significance and application prospects, especially in the rehabilitation field (Ang and Guan, 2013; Lebedev and Nicolelis, 2017) and as an alternative access method for physically disabled people (Gao et al., 2003; Pandarinath et al., 2017). The Steady-State Visual Evoked Potential (SSVEP) represents a stable neural response elicited by periodic visual stimuli, and its frequency tagging attribute can be leveraged in the BCI (Cheng et al., 2002; Norcia et al., 2015). Among a variety of BCI paradigms, the SSVEP-based BCI (SSVEP-BCI) has gained widespread attention due to its characteristics of non-invasiveness and high signal-to-noise ratio (SNR) and information transfer rate (ITR) (Bin et al., 2009; Chen et al., 2015a). Generally, the high-speed performance of the BCI is accomplished by a multi-target visual speller, which achieves a reportedly average online ITR of 5.42 bit per second (bps) (Nakanishi et al., 2018). Besides, the ease of use and significantly lower rate of the BCI illiteracy (Lee et al., 2019) make it a promising candidate for real-world applications.

In order to improve the performance of the BCI, rapid progress has been made to facilitate frequency recognition of the SSVEP (Zerafa et al., 2018). Based on whether a calibration or training phase is required for the extraction of spatial filters, the signal detection methods can be categorized into supervised methods and training-free methods. The supervised methods exploit an optimal spatial filter by a training procedure and achieve the state-of-the-art classification performance in the SSVEP-based BCI (Nakanishi et al., 2018; Wong et al., 2020a). These spatial filters or projection direction can be learned by exploiting individual training template (Bin et al., 2011), reference signal optimization (Zhang et al., 2013), inter-frequency variation (Yin et al., 2015), and ensemble reference signals (Nakanishi et al., 2014; Chen et al., 2015a) in the framework of canonical correlation analysis (CCA). Recently, the task-related components (Nakanishi et al., 2018) and the multiple neighboring stimuli (Wong et al., 2020a) have been utilized to derive spatial filters in order to boost the discriminative power of the learned model further. On the other hand, the training-free methods perform feature extraction and classification in one step without the training session in the online BCI. This line of work usually use a sinusoidal reference signal, and the detection statistics can be derived from the canonical correlation (Bin et al., 2009) and its filter-bank form (Chen et al., 2015b), noise energy minimization (Friman et al., 2007), synchronization index maximization (Zhang et al., 2014), and additional spectral noise estimation (Abu-Alqumsan and Peer, 2016).

Along with the rapid development of frequency recognition methods, continuous efforts have been devoted to share the SSVEP database (Bakardjian et al., 2010; Kolodziej et al., 2015; Kalunga et al., 2016; Kwak et al., 2017; Işcan and Nikulin, 2018) and contribute to public SSVEP database (Wang et al., 2017; Choi et al., 2019; Lee et al., 2019). Wang et al. (2017) benchmarked a 40-target database comprising 64-channel 5-s SSVEP trials of 35 subjects who performed the offline cue-spelling task in six blocks. Recently, Lee et al. (2019) have released a larger database of 54 subjects performing the 4-target offline and online task, and 62-channel 4-s SSVEP data were obtained having 50 trials per class. Choi et al. (2019) also provided a 4-target database, including physiological data and the 6-s SSVEP data which are collected from 30 subjects at three different frequency bands (low: 1–12 Hz; middle: 12–30 Hz; high: 30–60 Hz) during 2 days. Nevertheless, the number of public databases in the SSVEP-BCI community is still limited compared to other domains, such as computer vision, where a growing number of databases plays a critical role in the development of the discipline (Russakovsky et al., 2015). Compared to the other BCI paradigms, e.g., the motor imagery BCI, the SSVEP-BCI databases are also scarce (Choi et al., 2019). Therefore, more databases are need in the SSVEP-BCI field for the design and evaluation of methods.

To this end, we present a large **BE**nchmark database **T**owards SSVEP-BCI **A**pplication (BETA) in this study. The BETA database includes the data of 70 subjects performing the cued-spelling task. As an extension of the benchmark database (Wang et al., 2017), the number of targets is 40, and the frequency range is from 8 to 15.8 Hz. A key feature of the proposed BETA database is that it is developed for real-world applications. Different from the benchmark database, the BETA consists of the data collected outside the laboratory setting of the electromagnetic shielding room. Since it is imperative to reduce the calibration time from a practical perspective, the number of blocks is set to four instead of six that are used in the benchmark. A QWERT virtual keyboard is presented in flickers to approximate the conventional input device better and enhance user experience. To the best of our knowledge, so far, the BETA database has the largest number of subjects for the SSVEP-BCI. Since a larger database can capture the inter-subject variability better, the BETA database makes it possible to reflect a more realistic EEG distribution and potentially meet the demands of real-world BCI applications.

The remaining of the paper is organized as follows. First, the data acquisition and curation procedures are presented in section 2. The data record and availability are described in section 3. In section 4, data validation is performed, and 11 frequency recognition methods are compared on BETA. We discuss additional findings from the database in section 5. Finally, the conclusions are given in section 6.

## 2. Materials and Methods

### 2.1. Participants

Seventy healthy volunteers (42 males and 28 females) with an average age of 25.14 ± 7.97 (mean ± standard deviation, ranging from 9 to 64 years) participated in our study. All the participants had a normal or corrected to normal vision, and they all signed a written consent before the experiment; for the participants under 16 years old, the consent was signed by their parents. The study was carried out in accordance with the Declaration of Helsinki, and the protocol was approved by the Ethics Committee of Tsinghua University (No. 20190002).

### 2.2. Recruitment and Inclusion Criteria

Participants were recruited on a national scale to take part in the Brain-Computer Interface 2018 Olympics in China. The competition was held to contest and award individuals with a high performance of the BCI (SSVEP, P300, and Motor Imagery). The 70 participants who participated in this study have also participated in the second round of the contest (SSVEP-BCI track), and none of them was naive to the SSVEP-BCI. Before the enrollment, participants were informed that the data would be used in non-commercial scientific research. Participants who conformed to the experimental rules in the first round and were available for the second round planed by the contest schedule were included in the second round. All the participants met the following criteria: (1) they had no history of epileptic seizures or other neuropsychiatric disorders, (2) they had no attention-deficit or hyperactivity disorder, and (3) they had no history of brain injury or intracranial implantation.

### 2.3. Visual Speller

**Figure 1:**
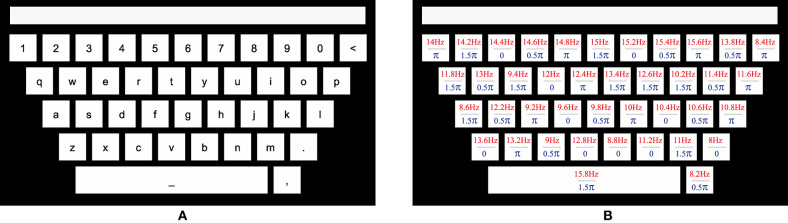
The QWERT virtual keyboard for a 40-target BCI speller. **(A)** The layout of a conventional keyboard with ten numbers, 26 alphabets and four non-alphanumeric keys (dot, comma, backspace <, and space _) aligned in five rows. The upper rectangle is designed to present the input character. **(B)** The frequency and initial phase of each target are encoded using the joint frequency and phase modulation.

This study designed a 40-target BCI speller for visual stimulation. In order to improve user experience, a graphical interface was designed to resemble the traditional QWERT keyboard. The virtual keyboard was presented on a 27-inch LED monitor (ASUS MG279Q Gaming Monitor, 1,920 × 1,080 pixels) with a refresh rate of 60 Hz. As illustrated in Figure 1A, 40 targets, including 10 numbers, 26 alphabets, and 4 non-alphanumeric signs (dot, comma, backspace < and space _) were aligned in five rows, with a spacing of 30 pixels. The stimuli had the dimension of 136 × 136 pixels (3.1° × 3.1°) for the square, and 966 × 136 pixels (21° × 3.1°) for the space rectangle. The topmost blank rectangle was for result feedback (Figure 1A).

A sampled sinusoidal stimulation method (Manyakov et al., 2013; Chen et al., 2014) was adopted to present the visual flicker on the screen. In general, the stimulus sequence of each flicker can be generated by

(1) $$\begin{array}{l} s\left(f,\phi ,i\right)=\frac{1}{2}\left\{1+\text{sin}\left[2\pi f\left(i/RefreshRate\right)+\phi \right]\right\} \end{array}$$

where *i* denotes the frame index in the stimulus sequence, and *f* and ϕ denote the frequency and phase values of the encoded flicker that uses a joint frequency and phase modulation (JFPM) (Chen et al., 2015a). The grayscale value of the stimulus sequence ranges from 0 to 1, where 0 indicates dark, and 1 indicates the highest luminance of the screen. For the 40 targets, the tagged frequency and phase values can be respectively obtained by

(2) $$\begin{array}{r} f_{k}=f_{0}+\left(k-1\right)\cdot \Delta f\\ \Phi_{k}=\Phi_{0}+\left(k-1\right)\cdot \Delta \Phi \end{array}$$

where the frequency interval Δ*f* is 0.2 Hz, the phase interval ΔΦ is 0.5 π, and *k* denotes the index from dot, comma, and backspace, followed by a to z and 0–9, and space. In this work, *f*_0_ and Φ_0_ are set to 8 Hz and 0, respectively. The parameters of each target are presented in Figure 1B. The stimulus was presented by MATLAB (MathWorks, Inc.) using Psychophysics Toolbox Version 3 (Brainard, 1997).

### 2.4. Procedure

This study includes four blocks of online BCI experiments with a cued-spelling task. The experiments were as follows. Each block consisted of 40 trials, and there was one trial for each stimulus target in a randomized order. Trials began with a 0.5 s cue (a red square covering the target) for gaze shift, which was followed by flickering on all the targets, and ended with a rest time of 0.5 s. The participants were asked to avoid eye blinking during the flickering process. During the 0.5 s rest, the resulting feedback, which represented one of the recognized characters, was presented in the topmost rectangle after online processing by a modified version of the FBCCA method (Chen et al., 2015b). For the first 15 participants (S1–S15), the flickering lasted at least 2 s, and for the remaining 55 participants (S16–S70), it lasted at least 3 s. In order to avoid visual fatigue, there was a short break between two consecutive blocks.

### 2.5. Data Acquisition

The 64-channel EEG data were recorded by SynAmps2 (Neuroscan Inc.) according to the international 10-10 system. The sampling rate was set 1,000 Hz, and the pass-band of the hardware filter was 0.15–200 Hz. A built-in notch filter was applied to remove the 50 Hz power-line noise. The event triggers were sent from the stimulus computer to the EEG amplifier and synchronized to the EEG data by a parallel port as an event channel. The impedance of all the electrodes was kept below 10 kΩ. The vertex electrode Cz was used as a reference. During the online experiment, nine parietal and occipital channels (Pz, PO3, PO5, PO4, PO6, POz, O1, Oz, and O2) were selected for online analysis to provide the feedback result. In order to record the EEG data in real-world scenarios, the data were recorded outside the electromagnetic shielding room.

### 2.6. Data Preprocessing

According to the previous study (Chen et al., 2015a,b), the SSVEP harmonics in this paradigm have a frequency range of up to around 90 Hz. Based on the finding, a band-pass filtering (i.e., zero-phase forward and reverse filtering using eegfilt in EEGLAB (Delorme and Makeig, 2004) between 3 and 100 Hz was conducted to remove the environmental noise. Then, the epochs were extracted from each block, and they included 0.5 s before the stimulus onset, 2 s (for S1–S15) or 3 s (for S16–S70) of the stimulation, and 0.5 s after the simulation. The last 0.5 s of the epochs could contain the SSVEP data if the duration of the trial was > 2 s (for S1–S15) or 3 s (for S16–S70). Since frequency resolution could not affect the classification result of the SSVEP (Nakanishi et al., 2017), all the epochs were then down-sampled to 250 Hz.

### 2.7. Metrics

The SSVEP data quality was evaluated quantitatively by the signal-to-noise ratio (SNR) analysis and classification analysis. As for the SNR-based analysis, in most of the previous studies (Chen et al., 2015a,b; Xing et al., 2018), the narrow-band SNR metric was used. The narrow-band SNR (in decibels, dB) can be defined as a ratio of the spectral amplitude at the stimulus frequency to the mean value of the ten neighboring frequencies (Chen et al., 2015b)

(3) $$\begin{array}{l} SNR=20{\text{log}}_{10}\frac{y\left(f\right)}{\sum_{k=1}^{5}\left[y\left(f-\Delta f\cdot k\right)+y\left(f+\Delta f\cdot k\right)\right]} \end{array}$$

where *y*(*f*) denotes the amplitude spectrum at frequency *f* calculated by the Fast Fourier Transform (FFT), and Δ*f* denotes the frequency resolution.

Along with the narrow-band SNR, we used the wide-band SNR as a primary metric to characterize better both the wide-band noise and the contribution of harmonics to the signals. The wide-band SNR (in decibels, dB) can be defined as:

(4) $$\begin{array}{l} SNR=10{\text{log}}_{10}\frac{\sum_{k=1}^{k=Nh}P\left(k\cdot f\right)}{\sum_{f=0}^{f=f_{s}/2}P\left(f\right)-\sum_{k=1}^{k=Nh}P\left(k\cdot f\right)} \end{array}$$

where *Nh* denotes the number of harmonics, *P*(*f*_*n*_) denotes the power spectrum at frequency *f*, and *f*_*s*_/2 represents the Nyquist frequency. In the wide-band SNR, the sum of power spectrum of multiple harmonics (*Nh* = 5) is regarded as the signal and the energy of full spectral band subtracted from the signal is considered as noise.

The classification accuracy and the information transfer rate (ITR) have been widely used in the BCI community to evaluate the performance of different subjects and algorithms. The ITR (in bits per min—bpm) can be obtained by (Wolpaw et al., 2002):

(5) $$\begin{array}{l} ITR=60\cdot \left({\text{log}}_{2}M+P{\text{log}}_{2}P+\left(1-P\right){\text{log}}_{2}\frac{1-P}{M-1}\right)/T \end{array}$$

where *M* denotes the number of classes, *P* denotes the classification accuracy, and *T* (in seconds) denotes the average target selection time. The variable *T* in the equation represents the sum of gaze time and overall gaze shift time. To calculate the theoretical ITR for offline analysis, a gaze shift time of 0.55 s is chosen according to the previous studies (Chen et al., 2015b; Wang et al., 2017), which was proven sufficient in an online spelling task (Chen et al., 2015b).

### 2.8. Statistical Analysis

A linear regression was conducted to understand the relationship between the SNR and ITR metrics. To meet the assumptions of linear regression, the following procedures were conducted. A scatter plot of SNR against ITR was diagrammed to establish the linearity by visual inspection. The independence of residuals was ascertained by using the Durbin-Watson test. The standardized residuals were checked in the range of ±3 to ensure that there were no outliers in the data. The homoscedascity was ensured by assessing a plot of standardized residuals versus standardized predicted values. The normality of residuals was guaranteed by assessing a normal probability plot. The *R*^2^ and adjusted *R*^2^ were calculated to reflect the goodness-of-fit of the regression model. The statistical significance of the model is evaluated by analysis of variance (ANOVA).

The ITR values obtained from different methods were compared using a one-way repeated-measures ANOVA with a within-subject factor of method. A Greenhouse-Geisser correction was applied if the sphericity was violated, as assessed by Mauchly's test of sphericity. When there was a significant main effect (*p* < 0.05), *post-hoc* paired-sample *t*-tests were performed and Bonferroni adjustment was applied for multiple comparison. To reflect the effect size, partial eta-squared (η^2^) was calculated. A Mann-Whitney U test was conducted to determine if there were differences in the SNR metrics. All the statistical procedures were processed using SPSS Statistics 20 (IBM, Armonk, NY, USA). Data were presented as mean ± standard error of the mean (s.e.m.) unless otherwise stated.

## 3. Record Description

The database used in this work is freely available for scientific research, where it is stored in the MATLAB .mat format. This database contains 70 subjects, and each subject corresponds to one mat file. The names of subjects are mapped to indices from S1 to S70 for de-identification. Each file in the database consists of a MATLAB structure array, which included the 4-block EEG data and its counterpart supplementary information as its fields. The website for accessing the database is http://bci.med.tsinghua.edu.cn/download.html.

### 3.1. EEG Data

After data preprocessing, the EEG data were store as a 4-way tensor, with a dimension of channel × time point × block × condition. Each trial included the 0.5-s data before the event onset, and the 0.5-s data after the time window of 2 or 3 s. For S1–S15, the time window was 2 s, and the trial length was 3 s, whereas for S16–S70, the time window was 3 s and the trial length was 4 s. Additional information about the channel and condition can be found in the following section about the supplementary information.

### 3.2. Supplementary Information

The supplementary information is comprised of personal information, channel information, BCI quotient, SNR, sampling rate, and each condition's frequency and initial phase. The personal information contained information about the age and gender of a subject. The channel information denoted a location matrix (64 × 4), where the first column represented the channel index, and the second and third columns represented the degree and radius in polar coordinates, respectively; and the last column represented the channel name. The SNR information consisted of the mean narrow-band SNR and wide-band SNR values of each subject, which were calculated by Equations (3) and (4), respectively. The initial phase was given in radius.

## 4. Data Evaluation

### 4.1. Temporal, Spectral, and Spatial Analysis

**Figure 2:**
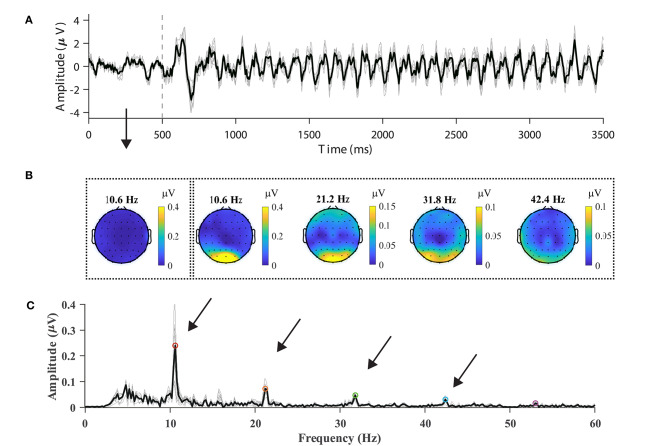
Typical SSVEP features in the temporal, spectral, and spatial domains. **(A)** Time course of average 10.6-Hz SSVEP of nine parietal and occipital channels (Pz, PO3, PO5, PO4, PO6, POz, O1, Oz, and O2). The dash line represents stimulus onset. **(B)** The topographic maps of SSVEP amplitudes at frequencies in the range from the fundamental signal (10.6 Hz) to the fourth harmonic (21.2, 31.8, and 42.4 Hz). The leftmost scalp map indicates the spectral amplitude at the fundamental frequency before stimulus. **(C)** The amplitude spectrum of the SSVEP of the nine channels at 10.6 Hz. Up to five harmonics are visible in the amplitude spectrum. The averaged spectrum across channels is represented in the dark line in **(A,C)**.

In order to validate the data quality by visual inspection, nine parietal and occipital channels (Pz, PO3, PO5, PO4, PO6, POz, O1, Oz, and O2) were selected, and epochs were averaged with respect to the channels, blocks, and subjects. For the sake of consistency regarding the data format, the subjects from S16 to S70 were chosen for analysis. Figure 2A illustrates the averaged temporal amplitude at the stimulus frequency of 10.6 Hz. After a delay, which was in the range of 100–200 ms, at the stimulus onset, a steady-state and time-locked characteristic could be observed in the temporal sequence, as shown in Figure 2A. The data between 500 and 3,500 ms were extracted and padded with 2,000 ms zeros, yielding a 0.2 Hz spectral resolution, as shown in Figure 2C. In the amplitude spectrum, the fundamental frequency (10.6 Hz: 0.266 μV) and three harmonics (21.2 Hz: 0.077 μV, 31.8 Hz: 0.054 μV, 42.4 Hz: 0.033 μV) could be distinguishable from the background EEG. Note that at high frequencies (> 60 Hz), the amplitude of both the harmonic signals and noise was small due to the volume conduct effect (van den Broek et al., 1998), which is why they are not shown in Figure 2C.

**Figure 3:**
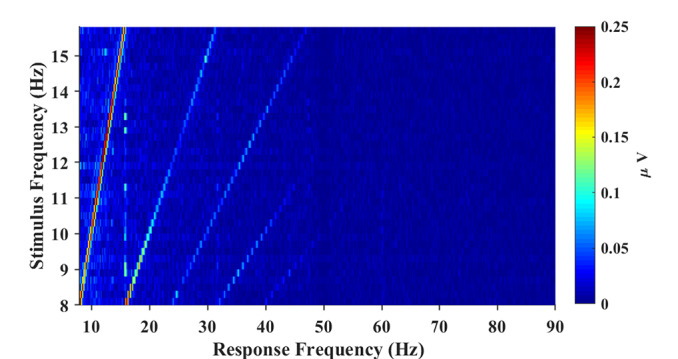
The amplitude spectrum as a function of stimulus frequency (frequency range: 8–15.8 Hz; frequency interval: 0.2 Hz). The spectral response of SSVEP decreases rapidly as the number of harmonics increases and up to 5 harmonics are visible from the figure.

Figure 2B illustrates the topographic mappings of the spectrum at frequencies in the range from the fundamental signal to the fourth harmonic. The result presented in Figure 2B indicates that fundamental and harmonic signals of the SSVEP are distributed predominantly in the parietal and occipital regions. The frontal and temporal regions of the topographic maps also show an increase in the spectrum, which can represent noise or SSVEP oscillation from the occipital region (Thorpe et al., 2007; Liu et al., 2017). In order to characterize the response property of the SSVEP, the amplitude spectrum is represented as a function of stimulus frequency in Figure 3. According to the amplitude spectrum, the spectral response of the SSVEP decreased rapidly with the number of harmonics; namely, up to five harmonics are visible. A dark line at the response frequency of 50 Hz results from the notch filtering. A bright line at the 15.8 Hz response frequency can be distractor stimulus from the SPACE target with a larger size.

### 4.2. SNR Analysis

**Figure 4:**
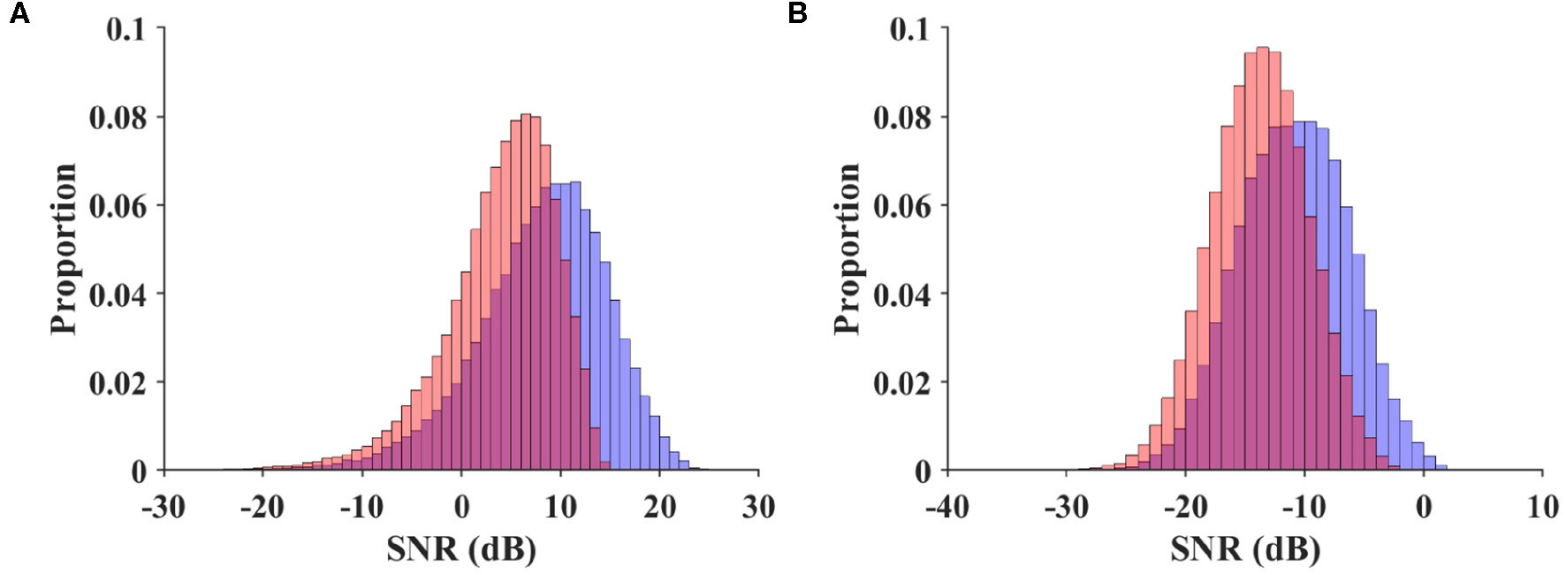
Normalized histogram of narrow-band SNR **(A)** and wide-band SNR **(B)** for trials in the benchmark database and BETA. The red diagram indicates the BETA, and the blue diagram indicates the benchmark database.

As a metric independent of different classification algorithms, the SNR measures available stimulus-evoked components in the SSVEP spectrum. In the SNR-based analysis, the BETA database was compared with the benchmark database of the SSVEP-based BCI (Wang et al., 2017). The narrow-band and wide-band SNR values were calculated for each trial by Equations (3) and (4), respectively. For a valid comparison, the EEG data in the benchmark database were band-pass filtered between 3 and 100 Hz (eegfilt in EEGLAB) before epoching. Trials in this database were padded with zeros (3 s for S1–S15, and 2 s for S16–S70) to provide a spectral resolution of 0.2 Hz. Figure 4 illustrates the normalized histogram of the narrow-band (Figure 4A) and wide-band SNRs (Figure 4B) for the trials in the two databases. For the narrow-band SNR, the BETA database had a significantly lower SNR (3.996 ± 0.018 dB) than the benchmark database (8.157 ± 0.024 dB), with a *p*-value of < 0.001, *z* = −142.212, Mann-Whitney *U*-test. Similarly, the wide-band SNR of the BETA database (−13.779 ± 0.013 dB) was significantly lower than the benchmark database (−10.918 ± 0.017 dB), with a p-value of < 0.001, *z* = −121.571, Mann-Whitney *U*-test. This was due in part to the individual differences in the SNR values of the two studies and in part because the EEG data were recorded outside the electromagnetic shielding room in the BETA database. The comparable results of the two SNR values also demonstrate the validity of the wide-band SNR metric that takes into account additional information on the wide-band noise and harmonics.

**Figure 5:**
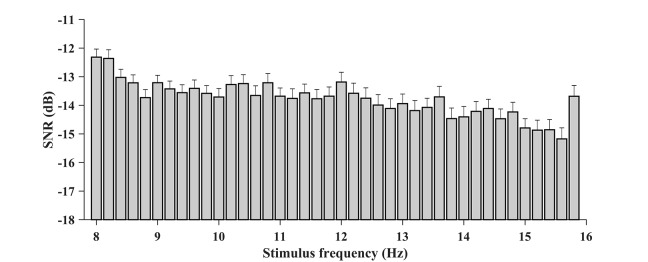
The wide-band SNR corresponding to the 40 stimulus frequencies (from 8 to 15.8 Hz with an interval of 0.2 Hz). A general declining tendency of SNR with the stimulus frequency can be observed. The SNR is higher at 15.8 Hz presumably because the target has a larger shape of the region.

In addition, the characteristics of SNR were analyzed with respect to each stimulus frequency. For the BETA database, the wide-band SNRs were calculated for the zero-padded trials, and the SNR associated with each condition was obtained by averaging the values per block and per person. Figure 5 illustrates the wide-band SNR corresponding to the 40 stimulus frequencies. In general, a declining tendency in SNR can be observed as the stimulus frequency increases. However, at some stimulus frequencies, e.g., 11.6, 10.8, 12, and 9.6 Hz, the SNR bumps up compared to their adjacent frequencies. Specifically, the average SNR value at 15.8 Hz was elevated by 1.49 dB compared to 15.6 Hz, which presumably was due in part to the larger region of visual stimulation.

### 4.3. Phase and Visual Latency Estimation

In order to further compare the BETA database with the benchmark database in Wang et al. (2017), we estimated the phase and visual latency of the BETA database. Nine consecutive stimulus frequencies in the first row of the keyboard were selected, and the SSVEP from the Oz channel (70 subjects) was extracted for analysis. The comparison procedure was performed according to that in the previous study (Wang et al., 2017) using a linear regression between the estimated phase and stimulus frequency (Russo and Spinelli, 1999). The visual latency for each subject using the slope *k* of the linear regression is obtained as follows:

(6) $$\begin{array}{l} Latency=-500\cdot k \end{array}$$

**Figure 6:**
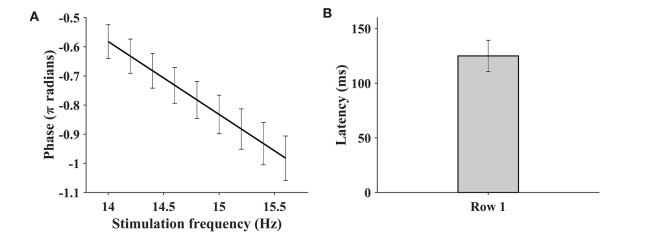
The phase as a function of stimulus frequency **(A)** and the bar plot of estimated latencies **(B)**. The SSVEP of Oz channel at nine consecutive stimulus frequencies (row 1 of the keyboard) is extracted for the purpose of analysis. The error bar indicates the standard deviation.

Figure 6 illustrates the phase as a function of the stimulus frequency, and the bar plot of the estimated latencies estimated by (6). The mean estimated visual latency was 124.96 ± 14.81 ms, which was close to 136.91 ± 18.4 ms of the benchmark database (Wang et al., 2017) and approximated to 130 ms. Therefore, a 130-ms latency was added to the SSVEP epochs for the subsequent classification analysis.

### 4.4. Accuracy and ITR on Various Algorithms

In this study, 11 frequency recognition methods, including six supervised methods and five training-free methods, were adopted to evaluate the BETA database. For S1–S15, the epoch length of 2 s was used for analysis, and for S16–S70, the epoch length was 3 s. A sliding window from the stimulus onset (latency corrected) with an interval of 0.2 s was applied to the epochs for offline analysis.

#### 4.4.1. Supervised Methods

**Figure 7:**
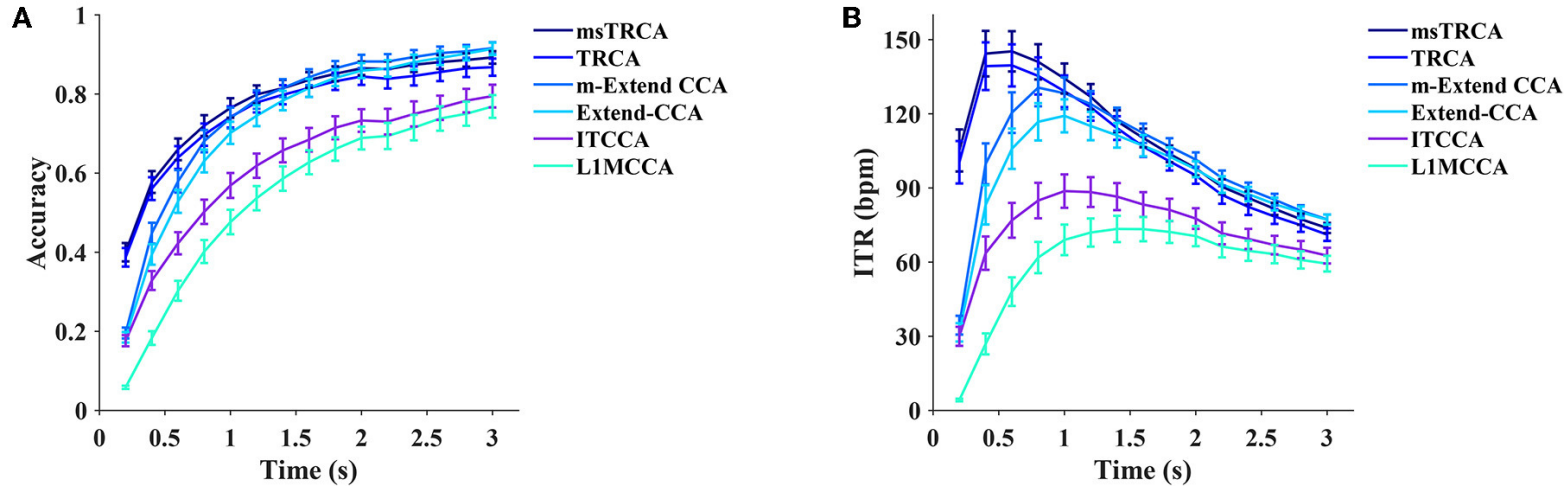
The average classification accuracy **(A)** and ITR **(B)** for six supervised methods (msTRCA, TRCA, m-Extended CCA, Extended CCA, ITCCA, and L1MCCA). Ten data lengths ranging from 0.2 to 3 s with an interval of 0.2 s were used for evaluation. The gaze shift time used the calculation of ITR was 0.55 s.

We choose six supervised methods, including the task-related component analysis (TRCA, Nakanishi et al., 2018), multi-stimulus task-related component analysis (msTRCA, Wong et al., 2020a), Extended CCA (Nakanishi et al., 2014), modified Extended CCA (m-Extended CCA, Chen et al., 2015a), L1-regularized multiway CCA (L1MCCA, Zhang et al., 2013), and individual template-based CCA (ITCCA, Bin et al., 2011) for comparison. The leave-one-out procedure on four blocks was applied to each subject to calculate the accuracy and ITR. Figure 7 illustrates the average accuracy and the ITR of the supervised methods. The results showed that the msTRCA outperformed other methods at data lengths < 1.4 s, and the m-Extended CCA achieved the highest performance at data lengths from 1.6 to 3 s. The one-way repeated measures ANOVA revealed that there were significant differences between the methods in the ITRs for all time windows. Specifically, for a short time window of 0.6 s, the main effect of methods showed there was a statistically significant difference in ITR, *F*_(1.895,130.728)_ = 186.528, *p* < 0.001, partial η^2^ = 0.730. *Post-hoc* paired *t*-tests showed that the order was as follows: msTRCA > TRCA > m-Extended CCA > Extended CCA > ITCCA > L1MCCA in ITR, where “>” indicates *p* was < 0.05 in the ITR with Bonferroni correction for pairwise comparison between the two sides. For a medium-length time window of 1.2 s, the main effect of methods showed there was a statistically significant difference in ITR, *F*_(1.797,124.020)_ = 197.602, *p* < 0.001, partial η^2^ = 0.741. *Post-hoc* paired *t*-tests showed the following: msTRCA / m-Extended CCA / TRCA > Extended CCA > ITCCA > L1MCCA (msTRCA vs m-Extended CCA: *p* = 0.678; m-Extended CCA vs TRCA: *p* = 1.000; Bonferroni corrected). The data length corresponding to the highest ITR varied between different methods; namely, the following results were achieved: msTRCA: 145.26 ± 8.15 bpm at 0.6 s, TRCA: 139.58 ± 8.52 bpm at 0.6 s, m-Extended CCA: 130.58 ± 7.53 bpm at 0.8 s, Extended CCA: 119.17 ± 6.67 bpm at 1 s, ITCCA: 88.72 ± 6.75 bpm at 1 s, L1MCCA: 73.42 ± 5.31 bpm at 1.4 s).

#### 4.4.2. Training-Free Methods

**Figure 8:**
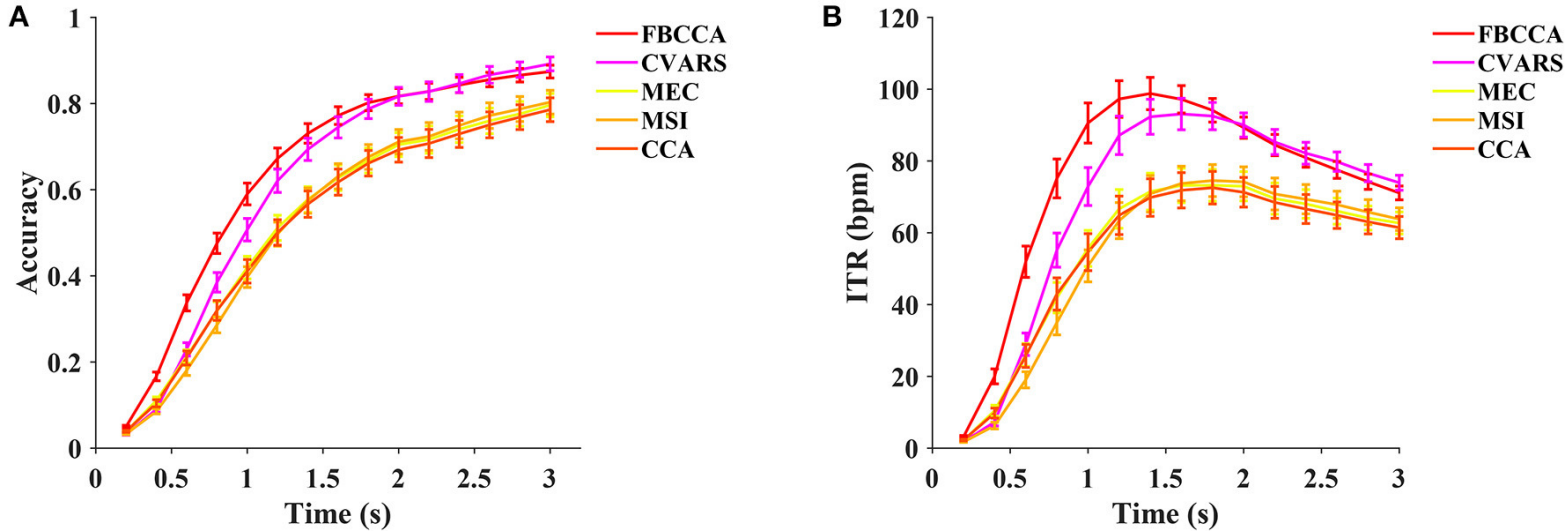
The average classification accuracy **(A)** and ITR **(B)** of five training-free methods (FBCCA, CVARS, MEC, MSI, and CCA). Ten data lengths ranging from 0.2 to 3 s with an interval of 0.2 s were used for evaluation. The gaze shift time used the calculation of ITR was 0.55 s.

In this study, five training-free methods, including the minimum energy combination (MEC, Friman et al., 2007), canonical correlation analysis (CCA, Bin et al., 2009), multivariate synchronization index (MSI, Zhang et al., 2014), filter bank canonical correlation analysis (FBCCA, Chen et al., 2015b), and canonical variates with autoregressive spectral analysis (CVARS, Abu-Alqumsan and Peer, 2016) are compared. As illustrated in Figure 8, the FBCCA was superior over the other methods at data lengths <2 s, and the CVARS outperformed the others at data lengths from 2 to 3 s. Significant differences in ITR were found between the methods by the one-way repeated measures ANOVA for all the data lengths. For a medium-length time window of 1.4 s, the main effect of methods showed there was a statistically significant difference in ITR, *F*_(1.876,129.451)_ = 79.227, *p* < 0.001, partial η^2^ = 0.534. *Post-hoc* paired *t*-tests with Bonferroni correction showed the following result: FBCCA > CVARS > CCA / MSI / MEC, *p* < 0.05 for all pairwise comparisons except CCA vs MSI (*p* = 1.000), CCA vs. MEC (*p* = 1.000), MSI vs. MEC (*p* = 1.000). As for the training-free methods, the highest ITR was achieved after 1.2 s, and the result was as follows: FBCCA: 98.79 ± 4.49 bpm at 1.4 s, CVARS: 93.08 ± 4.39 bpm at 1.6 s, CCA: 72.54 ± 4.54 bpm at 1.8 s, MSI: 74.54 ± 4.46 bpm at 1.8 s, MEC: 73.23 ± 4.43 bpm at 1.8 s.

Note that for the TRCA and msTRCA, the ensemble and filter-bank scheme were employed by default. Therefore, to ensure a fair comparison, the number of harmonics *N*_*h*_ was set to 5 in all the methods with sinusoidal templates except the m-Extended CCA according to Chen et al. (2015a) (*N*_*h*_ = 2). For all the methods without a filter bank scheme, the trials were band-pass filtered between 6 and 80 Hz except for the CVARS method, which was in line with the previous study (Nakanishi et al., 2015).

### 4.5. Correlation Between SNR and ITR

**Figure 9:**
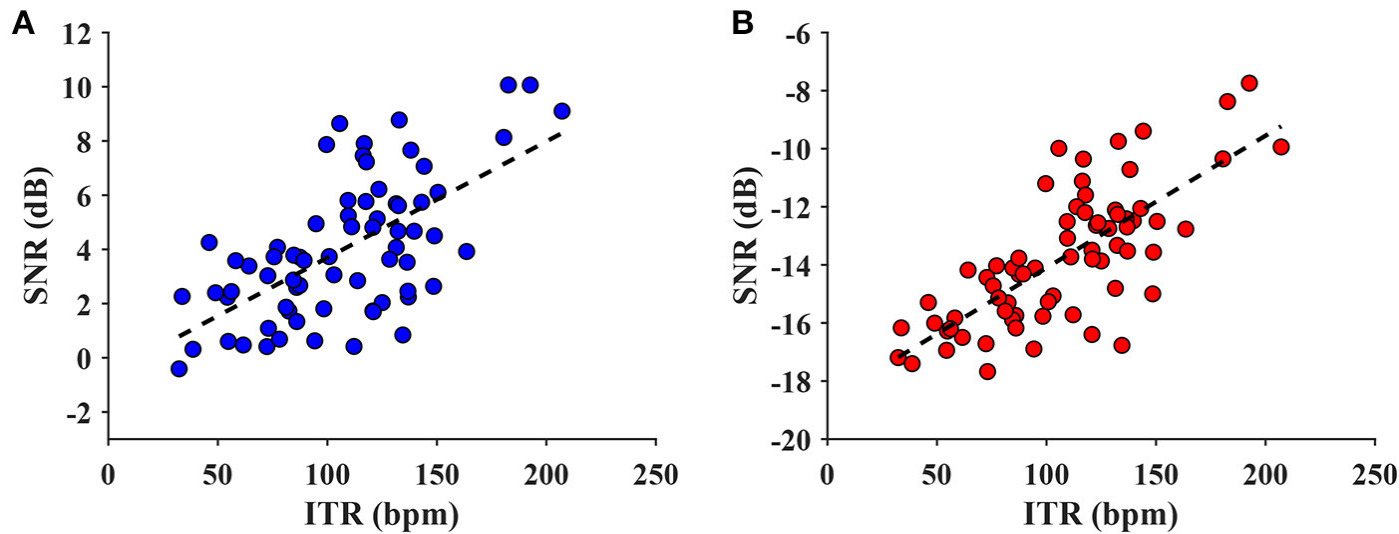
The scatter plot of narrow-band SNR vs. ITR **(A)** and wide-band SNR vs. ITR value **(B)**. The dash line indicates a linear model regressed on the data (**A**: adjusted *R*^2^ = 0.393, *p* < 0.001; **B**: adjusted *R*^2^ = 0.549, *p* < 0.001). The regression indicates that the wide-band SNR correlated better with the ITR than the narrow-band SNR.

In order to explore the relationship between the SNR and ITR metrics, the wide-band and narrow-band SNRs were both investigated. The maximum ITR for each subject (after averaging the ITR values by block) from the training-free FBCCA was chosen for the analysis. Figure 9 illustrates the scatter plots of the narrow-band and wide-band SNRs vs the ITR. As can be seen in Figure 9, the ITR was positively correlated with the SNR for both the narrow-band and wide-band values. For the narrow-band SNR, the statistical analysis reveals that the metric could significantly predict the ITR, *F*_(1,68)_ = 45.600, *p* < 0.001, and the narrow-band SNR accounted for 40.1% of the variation in the ITR with adjusted *R*^2^ = 0.393. The wide-band SNR could also statistically significantly predict the ITR, *F*_(1,68)_ = 84.944, *p* < 0.001, accounting for 55.5% of the variation in the ITR with adjusted *R*^2^ = 0.549. This result indicates that the metric of a wide-band SNR is more correlated with and can predict better ITR than a narrow-band SNR.

### 4.6. BCI Quotient

**Figure 10:**
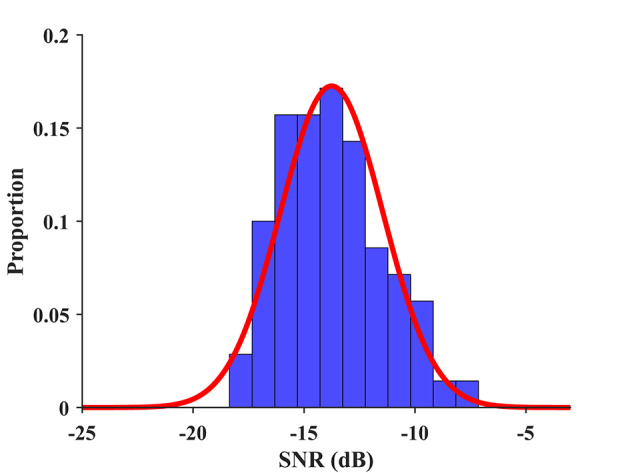
The distribution of wide-band SNR and its fitting to a normal distribution. An individual's SNR of the SSVEP is rescaled to the range of normal distribution by Equation (7) to obtain the BCI quotient.

The electroencephalographic signals, including the SSVEP showed individual differences in population. In this study, we propose a BCI quotient to characterize the subject's capacity to use the SSVEP-BCI measured at the population level. Equivalent to the scoring procedure of intelligence quotient (IQ) (Wechsler, 2008), the (SSVEP-) BCI quotient is defined as follows:

(7) $$\begin{array}{l} Quotient_{BCI}=15\cdot \frac{SNR-\mu }{\sigma }+100 \end{array}$$

where SNR represents the wide-band SNR, and the mean and standard deviation in this study are μ = −13.78 and σ = 2.31, respectively, as shown in Figure 10. The mean and standard deviation can be estimated more accurately for a larger database in the future. The BCI quotient rescales an individual's SNR of the SSVEP to the range of normal distribution N(100,15). Since the BCI quotient denotes a relative value derived from SNR, and SNR is correlated with the ITR, the BCI quotient has the potential to measure signal quality and performance for individuals in the SSVEP-BCI. Higher BCI quotient values indicate a higher probability of good BCI performance. For instance, the BCI quotients of S20 and S23 were 74.71 and 139.21, respectively, which reveals a prior to the individual level of the ITR, i.e., 73.09 bpm for S20 and 192.63 bpm for S23. The BCI quotients for each subject were listed in Table S1 and the result of a regression analysis between the BCI quotient and ITR was provided in the Supplementary Material.

## 5. Discussion

### 5.1. Data Quality and Its Applicability

Compared to the benchmark database (Wang et al., 2017), the BETA database had lower SNR and the corresponding ITR in the classification (for the benchmark database: FBCCA, 117.96 ± 7.78 bpm at 1.2 s; m-Extended CCA, 190.41 ± 7.90 bpm at 0.8 s; CCA, 90.16 ± 6.81 bpm at 1.6s; 0.55-s rest time for comparison; Chen et al., 2015b; Wang et al., 2017). This can be expected since, in BCI applications, neither there is actually electromagnetic shielding condition nor can be ensured that each subject has a high SNR of the SSVEP. The discrepancy in SNR was due in part to the distinct stimulus duration, which was 2 or 3 s for the BETA database and 5 s for the benchmark database. However, even at the same stimulus duration (a 3-s trial after stimulus onset for 55 subjects in the BETA and 35 subjects in the benchmark), the BETA database had significantly lower SNR than the benchmark database (narrow-band SNR: BETA 4.296 ± 0.021 dB, benchmark 5.218 ± 0.020 dB, *p* < 0.001, *z* = −34.039, Mann-Whitney *U*-test; wide-band SNR: BETA −13.531 ± 0.015 dB, benchmark −12.912 ± 0.015 dB, *p* < 0.001, *z* = −28.814, Mann-Whitney *U*-test). Therefore, the present BETA database poses challenges to the traditional frequency recognition methods and provides opportunities for the development of robust frequency recognition algorithms intended for real-world applications.

A large number of subjects in the BETA database has the merit of reducing the over-fitting and can provide an unbiased estimation in the evaluation of frequency recognition algorithms. Also, a large volume of the BETA provides an opportunity for the research on transfer learning for the purpose of exploiting common discriminative patterns across subjects. Note that in the BETA database, the number of blocks of each subject is smaller than that in the benchmark database. Since reducing the training and calibration time is critical for the BCI application, the proposed database can serve as a test-bed for the development of supervised frequency recognition methods based on smaller training samples or few-shot learning. It is noteworthy that the application scenario of the BETA database is not limited to the 40-target speller presented in the study. Namely, practitioners can select a subset of the 40 targets (e.g., 4, 8, 12 targets) and design customized paradigms to meet the requirements of a variety of real-world applications. However, since the paradigm of the BETA database falls into the category of dependent BCI where subjects were instructed to redirect their gaze during target selection, the gaze shifting limits its applicability for patient users challenged by oculomotor control. Specifically for these scenarios, gaze independent SSVEP-BCI that is based on covert selective attention (Kelly et al., 2005; Allison et al., 2008; Zhang et al., 2010; Tello et al., 2016) or stimulation via closed eyes (Lim et al., 2013; Hwang et al., 2015) could be deployed, although the information throughput is low with only 2 or 3 targets and modest accuracy. Nevertheless, the BETA database shows its potential to unlock new applications in SSVEP-BCI for alternative and augmentative communication. With the advent of big data, the BETA shows promise for facilitating brain modeling at a population level and help developing novel classification approaches or learning methodology, such as federated learning (Mcmahan et al., 2017) based on big data.

### 5.2. Supervised and Training-Free Methods

In general, the state-of-the-art supervised frequency recognition methods have the advantage of higher performance regarding the ITR, and the training-free methods excel in ease of use. In this study, two of the supervised methods (the m-Extended CCA, and the Extended CCA) outperformed the five training-free algorithms at all the data lengths. Specifically, at the short-time window (0.2–1 s) the supervised methods (the msTRCA, the TRCA, the m-Extended CCA, and the Extended CCA) outperformed the training-free methods by a large margin (see Figure S1). This was because the introduction of the EEG training template and the learned spatial filters facilitated the SSVEP classification. At the time window longer than 2 s (2.2–3 s), the *post hoc* paired t-tests showed that no significant difference is between the m-Extended CCA and the Extended CCA, between the FBCCA and the CVARS, and among the ITCCA, the CCA, the MEC, and the MSI (*p*>0.05, Bonferroni corrected). Such a result suggests certain common mathematical grounds shared by these algorithms in principle (Wong et al., 2020b). Interestingly, as reported in the previous study (Nakanishi et al., 2018), the TRCA method performance decreased presumably due to the lack of sufficient training block for subjects with low SNR. As evidenced by the previous study (Nakanishi et al., 2018), for the TRCA the number of training data greatly affects classification accuracy (≈ 0.85 with 11 training blocks and ≈ 0.65 with two training blocks for a 0.3-s time window). This implies that methods with a sinusoidal reference template (e.g., m-Extended CCA, Extended CCA, and FBCCA, etc.) may be more robust than those without it (Wong et al., 2020b). To sum up, the presented classification analysis demonstrates the utility of different competing methods on the BETA. Besides, the comparison of different methods on a single database complements the previous work of Zerafa et al. (2018), where the performance of various methods was not compared on the same database.

### 5.3. SNR Comparison

The SNR-based analysis results showed that the wide-band SNR was more correlated with the ITR than the narrow-band SNR. As shown in Figure 4, a transition from the narrow-band SNR to the wide-band SNR did not affect the relative relationship between the SNRs of the two databases. Nevertheless, the wide-band SNR metric reduces the skewness of data distribution from −0.708 to −0.081 for benchmark database, and from −1.108 to −0.142 for the BETA database; the narrow-band SNR was followed by the wide-band SNR, which makes the SNR characteristic be more likely to follow the Gaussian distribution. According to Parseval's theorem, the spectral power of a signal is equal to its power in the time domain, so the formulated wide-band SNR has equivalent mathematical underpinning as a metric of temporal SNR counterpart. Apart from its expressive power of wide-band SNR, this metric is also intuitive in the description of signal and noise due to the frequency tagging attribute of the SSVEP.

## 6. Conclusion

In this paper, a **BE**nchmark database **T**owards BCI **A**pplication (BETA) for the 40-target SSVEP-BCI paradigm is presented. The BETA database is featured by its large number of subjects and its paradigm that is well-suited for real-world applications. The quality of the BETA is validated by the typical temporal, spectral and spatial profile of the SSVEP, together with the SNR and the estimated visual latency. The BETA database compares eleven frequency recognition methods, including six supervised methods and five training-free methods. The result of classification analysis validates the data and demonstrates the performance of different methods in one arena as well. As for the metric to characterize the SSVEP, we recommend adopting the wide-band SNR at the single-trial level and use the BCI quotient at the population level. We expect the proposed BETA database can pave the way for the development of methods and paradigms for practical BCI and push the boundary of the BCI toward real-world application.

## Data Availability Statement

The datasets presented in this study can be found in online repositories. The names of the repository/repositories and accession number(s) can be found below: http://bci.med.tsinghua.edu.cn/download.html. The datasets has an alternative source for download at https://figshare.com/articles/The_BETA_database/12264401 for the sake of stable access.

## Ethics Statement

The studies involving human participants were reviewed and approved by the Ethics Committee of Tsinghua University. Written informed consent to participate in this study was provided by the participants, and where necessary, the participants' legal guardian/next of kin.

## Author Contributions

BL conducted the data curation and analysis and wrote the manuscript. XH designed the paradigm and performed the data collection. YW and XC performed the data collection and revised the manuscript. XG supervised the study. All authors contributed to the article and approved the submitted version.

## Conflict of Interest

The authors declare that the research was conducted in the absence of any commercial or financial relationships that could be construed as a potential conflict of interest.
