# Supplementary material for: BETA: A Large Benchmark Database Toward SSVEP-BCI Application
Source: Front Neurosci. 2020 Jun 23;14:627. doi: 10.3389/fnins.2020.00627 (PMC7324867; doi:10.3389/fnins.2020.00627)
Supplement: Supplementary file 1 [file Data_Sheet_1.pdf]

Supplementary Material

1 SUPPLEMENTARY FIGURE

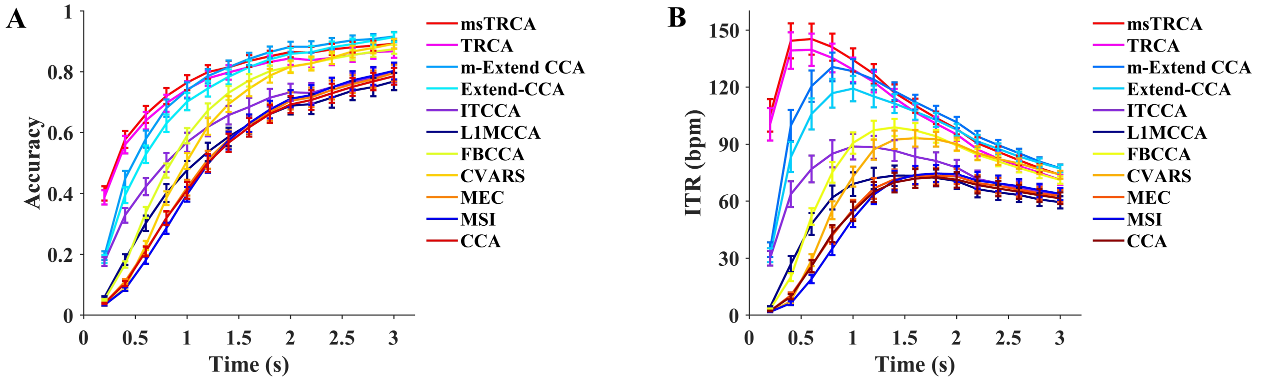

Figure S1. The average classification accuracy **(A)** and the ITR **(B)** for 11 frequency recognition methods (msTRCA, TRCA, m-Extended CCA, Extended CCA, ITCCA, L1MCCA, FBCCA, CVARS, MEC, MSI and CCA). Ten data lengths ranging from 0.2 s to 3 s with an interval of 0.2 s were used for evaluation. The gaze shift time used the calculation of ITR was 0.55 s.

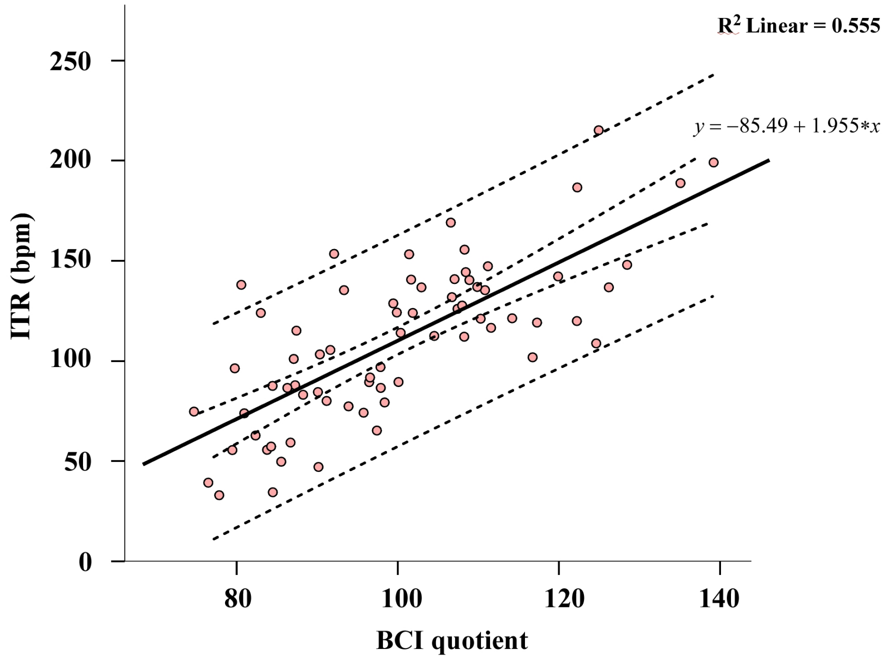

Figure S2. The scatter plot of BCI quotient vs ITR value **(B)**. The thick line indicates a linear model regressed on the data ($R^2 = 0.555$, $p < 0.001$). The prediction equation has the form $y = -85.49 + 1.955x$. The curved dashed line and the straight dashed line represent confidence interval and prediction interval, respectively.

2 LINEAR REGRESSION BETWEEN BCI QUOTIENT AND ITR

A linear model regressing the ITR on the BCI quotient was hereby performed, as illustrated in Figure S2. The confidence and prediction interval were plotted as the curved dashed line and the straight dashed line, respectively. The statistical analysis indicated that the BCI quotient could significantly predict the ITR, $F(1,68) = 84.923, p < 0.001$, and it accounted for 55.5% of the variation in the ITR with adjusted $R^2 = 0.549$. The prediction equation has the form

$$y = -85.49 + 1.955x \tag{S1}$$

where x and y represent the BCI quotient and the ITR, respectively. This implied that there was a predicted increase in ITR of 1.955 (95% CI, 1.531 to 2.378) bpm for every extra one score of the BCI quotient on average. The coefficient and the intercept of the model were both statistically significant ($p < 0.001$). To guarantee the extrapolation is valid in application, the x should be greater than 43.73 to satisfy $y > 0$.

3 SUPPLEMENTARY TABLE

Table S1: Individual BCI quotient and ITR.

Subject ID	BCI quotient	ITR (bpm)
1	108.44	139.63
2	92.10	148.49
3	111.19	143.02
4	87.09	98.37
5	80.58	134.44
6	87.26	85.97
7	83.00	120.77
8	96.45	87.48
9	102.94	132.28
10	98.37	77.22
11	77.84	32.30
12	101.66	136.97
13	99.44	125.03
14	91.63	103.00
15	93.33	131.44
16	97.86	94.84
17	79.47	54.38
18	135.09	182.66
19	122.23	116.83
20	74.71	73.09
21	108.89	136.39
22	107.42	122.74
23	139.21	192.63
24	104.53	109.57
25	124.63	105.62
26	97.89	84.67

27	101.86	120.75
28	117.29	116.35
29	108.25	109.42
30	114.20	117.79
31	97.41	64.19
32	80.95	72.39
33	82.35	61.61
34	106.72	128.43
35	99.88	120.96
36	110.83	131.44
37	101.41	148.84
38	95.76	72.80
39	90.33	100.83
40	100.09	87.37
41	76.48	38.64
42	126.20	132.80
43	90.09	82.46
44	90.16	46.05
45	79.77	94.24
46	86.30	84.49
47	86.69	58.15
48	128.47	144.17
49	122.28	180.56
50	93.89	75.67
51	116.74	99.62
52	107.05	136.78
53	91.17	78.14
54	84.46	86.02
55	84.49	33.68
56	119.90	138.09
57	109.87	132.46
58	111.58	113.79
59	83.78	54.76
60	100.36	111.07
61	85.54	48.94
62	96.56	89.35
63	106.58	163.57
64	88.25	81.16
65	84.28	56.19
66	108.29	150.49
67	124.94	207.16
68	110.29	117.60
69	87.42	112.14
70	107.99	123.50
